# Presence of Emerging Contaminants Upstream and Downstream of an Urban Wastewater Treatment Plant

**DOI:** 10.3390/toxics14050402

**Published:** 2026-05-07

**Authors:** Kyla Charlebois, Eva N. Nyutu

**Affiliations:** Department of Biology, University of Detroit Mercy, 4001 W. McNichols Rd, Detroit, MI 48221, USA; charlekm@udmercy.edu

**Keywords:** emerging contaminants, great lakes, pharmaceuticals, pesticides, urban, wastewater

## Abstract

Several issues about the quality of urban surface waters, such as the Detroit River, are becoming a concern due to the increasing detection of emerging contaminants. Although the emerging contaminants are present in low concentrations—ranging from nanograms per liter (ng/L) to micrograms per liter (µg/L)—these raise serious concerns about long-term effects on human health and aquatic ecosystems, particularly when left unregulated. Municipal wastewater effluent has been reported as one of the major pathways for these emerging contaminants. Most treatment plants are not equipped to effectively remove many emerging contaminants, allowing them to enter surface waters. To assess the presence of these emerging contaminants, water samples were collected during the summer from sites near the upstream and downstream of the Detroit wastewater treatment plant. Among the sixteen emerging contaminants analyzed were pharmaceuticals, personal care products, and pesticides. Ten of these, such as sucralose, caffeine, acetaminophen, and bisphenol A, were detected at both locations, with concentrations ranging from 42 to 4100 ug/L. Elevated contaminant levels found downstream can come from various sources, such as agricultural runoff, leachate from landfills, overland flow, and Combined Sewer Overflows (CSOs). Furthermore, local pharmaceutical usage patterns and the effectiveness of our treatment facilities play significant roles in the contaminant concentrations we see. Tracking emerging contaminants both upstream and downstream of treatment plants is crucial for pinpointing vulnerable watersheds. This vital information enables us to establish a solid baseline and craft effective strategies to lower contaminant levels.

## 1. Introduction

The Great Lakes Basin contains about 84% of the United States’ fresh surface water and approximately 21% of the world’s. The Great Lakes Basin is considered essential to both the United States and Canada, providing drinking water to more than 30 million people as well as supporting major industries and vital natural resources [1]. The Great Lakes Basin provides economic stability for both countries; it is a source of fresh water, recreational activities, and global trade, contains a diverse aquatic ecosystem, and supports wastewater management [2,3,4]. For several decades, there has been extensive industrialization and urban development around the Great Lakes Basin, driven largely by increasing human populations [4,5]. One of the major surface waters in the Great Lakes Basin currently experiencing significant water quality issues is the Detroit River. The Detroit River is the main drinking water source for roughly 4.5 million residents in Detroit and Windsor (Canada) and has faced a lot of anthropogenic effects [2,4].

One of the main challenges is the increasing levels of emerging contaminants in the Detroit River, which include personal care products (PPCPs), endocrine-disrupting chemicals (EDCs), pesticides, and pharmaceuticals. Some pharmaceuticals and personal care products (PPCPs) also function as endocrine-disrupting chemicals (EDCs), leading to overlap between these categories [6,7]. Examples of PPCPs include prescription and over-the-counter medications for humans and animals, as well as chemicals used in livestock and poultry production [8]. Additionally, PPCPS have been reported to include most chemicals found in cosmetics, sunscreens, antifungals, insect repellents, and antibacterial agents such as triclosan and triclocarban [9,10]. For pharmaceuticals, this includes antibiotics, antidepressants, stimulants, X-ray contrast agents, anti-inflammatory drugs, lipid regulators, β-blockers, antihypertensives, and illicit or recreational drugs [10]. Pesticides include chemicals and compounds used in agricultural activities and pest management [2,4,5]. Many pesticides have been reported to contaminate water, air, and soil. Pesticides have been reported to mimic hormones found in people and usually affect the endocrine system [2,4,5,7]. Endocrine-disrupting chemicals (EDCs) have been shown to disrupt the development and reproductive systems of aquatic organisms [11,12].

Current studies have shown that the presence of emerging contaminants like PPCPs, EDCs, pesticides, and pharmaceuticals usually affect organisms in the aquatic ecosystems and people’s health [13,14,15]. One reported issue is the horizontal transfer of antibiotic resistance to humans. When antibiotics enter surface waters, it usually results in the development of new bacterial strains that are resistant to antibiotics [13,14]. Moreover, these emerging contaminants can disrupt the human endocrine system by affecting sexual hormone regulation and differentiation, embryonic development, and metabolic maturation. These disruptions can subsequently impair reproductive health, leading to conditions such as low sperm count and poor sperm quality [13,16,17].

The issue of emerging contaminants in the Detroit River is not yet well understood, as studies have reported mixed findings. One study examined emerging contaminants in the Lake Huron–Erie corridor, and identified 50 compounds and reported that their presence was linked to the proximity of Detroit wastewater treatment plants (DWWTPs) and other anthropogenic influences in the Detroit River basin [4,18]. Another study analyzed tissue samples from dreissenid mussels (zebra and quagga mussels; *Dreissena* spp.) and found that mussels located near waterways receiving runoff and non-point sources had higher contaminant concentrations than those near DWWTP discharges and combined sewer overflows (CSOs) [10]. Ref. [19] examined 51 emerging contaminants in the Detroit River and reported that 14 of these compounds were detected at higher concentrations in DWWTP effluent than in influent. Lastly, Ref. [20] investigated 200 emerging contaminants in tributaries of the Great Lakes Basin and similarly found higher concentrations in DWWTP influent than in effluent.

Most environmental agencies are not able to regulate and monitor emerging contaminants in wastewater treatment plants. This is largely due to the limited standardized analytical methods, which are often unable to detect these contaminants at low concentrations [2,10,14,21,22]. As a result, the potential risks associated with short- or long-term exposure to these contaminants at low concentrations ranging from micrograms per liter (µg/L, parts per billion) to nanograms per liter (ng/L, parts per trillion) remain poorly understood for both humans and aquatic organisms [2,4,13,22].

Several studies have reported that conventional wastewater treatment processes are not very effective atremoving emerging contaminants [2,3,8,23,24,25,26]. As a result, these contaminants can enter aquatic ecosystems as parent compounds, metabolites, or transformation products, while some may remain undegraded [2,4,10,14,21,23]. To identify the most effective removal strategies for emerging contaminants, researchers must understand their molecular properties. Various emerging contaminants characteristics like molecular size, charge, hydrophobicity, and polarity have been shown to affect how they are absorbed by membranes [2,4,10,14,21,23,27].

It is currently unclear whether the spatial distribution of emerging contaminants near the discharge points of the Detroit wastewater treatment plant (DWWTP) is confined to areas only a few meters away or whether these substances are more broadly dispersed throughout the surrounding surface waters. We hypothesize that both the frequency and concentration of these contaminants will be higher at downstream locations near the DWWTP, due to the continuous release of treated effluent and the facility’s close proximity to the Rouge River. Consequently, ongoing monitoring and assessment are essential to identify and distinguish emerging contaminants specifically originating from the DWWTP. Quantifying the levels of these substances will improve our understanding of their diversity, behavior, and concentrations within the current treatment processes. Such knowledge is critical for safeguarding the future ecological health and water quality of the aquatic environment.

## 2. Methods

### 2.1. Sampling Sites

**Figure 1:**
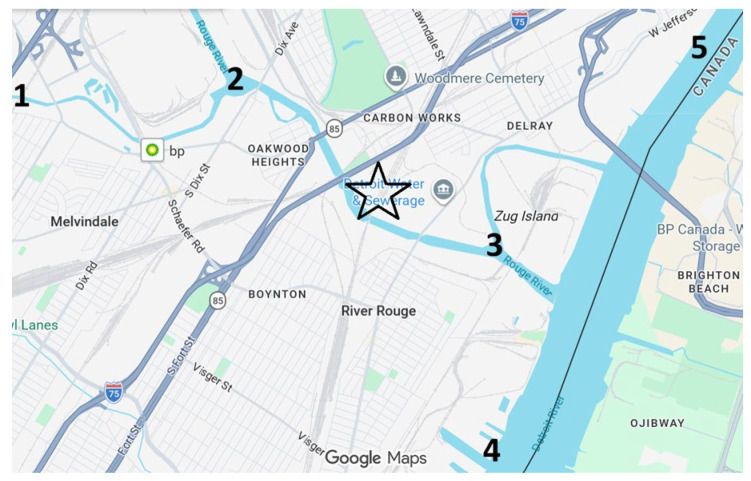
The star (★) marks the Detroit wastewater treatment plant (DWWTP), while Sites 1–5 represent sampling locations.

Samples were collected during the summer from five designated sites located upstream and downstream of the Detroit Wastewater Treatment Plant (DWWTP), near the Detroit River International Wildlife Refuge. The sampling locations were selected based on their proximity to key water management facilities, particularly the DWWTP as well as their ecological and recreational importance. Each site was assigned a numerical code 1–5 as follows: (refer to Figure 1). Site 1: Clinton River Mouth (42°35′40.7″ N, 82°46′01.6″ W), Site 2: Northeast Belle Isle (42°35′17.1″ N, 82°95′94.7″ W), Site 3: Southwest Belle Isle (42°33′35″ N, 82°98′64.2″ W), Site 4: Rouge River Mouth (42°27′52.1″ N, 83°11′07.6″ W), Site 5:Detroit River International Wildlife Refuge/Trenton Channel (42°09′97.8″ N, 83°18′26.0″ W) (Table 1).

### 2.2. Collection of Water Samples

Grab samples were collected using stainless steel containers from a depth of 0.5 m below the surface at locations upstream and downstream of the DWWTP, accessed by boat. Samples were transferred into pre-cleaned, unfiltered amber glass bottles and stored at 4 °C. During the summer, additional water samples were collected from Sites 1–5 using a 2 m polyethylene dipper. These sites, which were accessible only by boat due to water depth, were selected based on their proximity to the wastewater treatment plant and their ecological and recreational importance. To prevent cross-contamination, gloves were changed between sampling sites, and all equipment was disinfected between uses with a commercial detergent (Alconox®) powdered laboratory detergent was obtained from MilliporeSigma (Burlington, MA, USA) and thoroughly rinsed with distilled water. At each of the five sampling sites, we collected 10 water samples to ensure sufficient replication for reliable measurement of endocrine disruptors. The study was conducted during a single growing season, specifically in the summer. All samples were maintained at 4 °C and delivered to commercial laboratories for analysis within one hour of collection.

### 2.3. Physical—Chemical Parameters of Water Samples

Physicochemical parameters were measured at each sampling location, including water temperature (°C), total dissolved solids (TDS, ppm), pH, turbidity (NTU), and electrical conductivity (EC, µS/cm). These variables were assessed using a digital water-quality flow meter positioned at 60% of the total water-column depth. In addition, water temperature and depth were recorded at each site using an onboard GPS system (Garmin Ltd., Olathe, KS, USA). Precipitation data for the 30 days preceding each sampling event were obtained from the National Oceanic and Atmospheric Administration’s National Centres for Environmental Information, using records from the Detroit Metro Airport weather station (latitude: 42.2313; longitude: −83.3308) (Table 2).

### 2.4. Emerging Contaminants: Extraction and Analysis

Emerging contaminants were extracted using solid-phase extraction and analyzed by liquid chromatography tandem mass spectrometry (LC–MS/MS) in both positive and negative ionization modes. To evaluate analytical precision and accuracy, laboratory control samples (LCS) and corresponding duplicates were analyzed for each batch Weck Laboratories, Inc. City of Industry, California. Sample preparation followed the protocol outlined in U.S. EPA Method 3005A and adhered to the procedures described by [28].

### 2.5. Sample Preparation

Water samples were collected from Sites 1–5 using pre-cleaned amber glass containers, as previously described. Samples were kept on ice during transport to the laboratory. Upon arrival, they were acidified to a pH below 2 using concentrated sulfuric acid and stored at 4 °C until extraction. The use of sulfuric acid has been shown to effectively preserve the target analytes without degradation [28].

A 1000 mL aliquot of each sample was loaded onto Waters hydrophilic–lipophilic balance (HLB) solid-phase extraction cartridges at a flow rate of 15 mL/min. Prior to sample loading, cartridges were sequentially conditioned with dichloromethane, tert-butyl methyl ether (MTBE), methanol, and reagent-grade water. After sample loading, cartridges were rinsed with reagent-grade water and dried under a nitrogen stream for one hour. Target analytes were eluted first with methanol, followed by a 10:90 methanol–MTBE mixture. The combined eluates were then concentrated under nitrogen to a final volume of 1 mL.

### 2.6. Liquid Chromatography–Tandem Mass Spectrometry

All samples were analyzed by LC–MS/MS using electrospray ionization (ESI; positive and negative) or atmospheric-pressure chemical ionization (APCI; positive). The analytical system consisted of an autosampler (HTC-PAL, CTC Analytics, Zwingen, Switzerland) and a binary pump (Agilent, Palo Alto, CA, USA). Chromatographic separation was achieved on a Synergy Max-RP C12 column (250 × 4.6 mm, 4 µm; Phenomenex, Torrance, CA, USA). A binary gradient was employed using 0.1% formic acid in water (solvent A) and 100% methanol (solvent B), delivered at 700 µL/min. The gradient began with 5% B for 3.5 min, increased linearly to 80% B by 10 min, and was held for 3 min before transitioning to 100% B for 8 min. The system was then re-equilibrated at 5% B for 9 min, giving a total run time of 30 min per sample. The injection volume was 10 µL.

In addition to the emerging contaminants identified by [28], estrone and estriol were included in the analysis. Both compounds were analyzed using APCI in positive mode and exhibited precursor ions at *m*/*z* 271, representing the [M + H]+ ion for estrone and the dehydrated ion [M − H_2_O + H]+ for estriol. Both compounds also produced a product ion at *m*/*z* 253 due to the loss of water (18 Da). Despite identical ion transitions, chromatographic separation enabled clear differentiation of estrone and estriol. Instrument parameters were as follows: declustering potential, 46 V for estriol and 31 V for estrone; collision energy, 19 eV for estriol and 23 eV for estrone; and collision cell exit potential, 10 V for estriol and 16 V for estrone.

## 3. Results

The conductivity of wastewater samples varied across the sampling sites, with measurements of 435 μS/cm at Site 1, 929 μS/cm at Site 2, 526 μS/cm at Site 3, 288 μS/cm at Site 4, and 236 μS/cm at Site 5. Turbidity levels followed a similar pattern: Site 1 measured 208 ppm, Site 2 reached 676 ppm, Site 3 measured 378 ppm, Site 4 recorded 198 ppm, and Site 5 showed 170 ppm. In general, increases in conductivity were associated with higher turbidity levels across the sites. The pH of the wastewater samples ranged from 7.8 to 9.0, and temperatures varied between 17.9 and 29 °C (Table 3).

**Figure 2:**
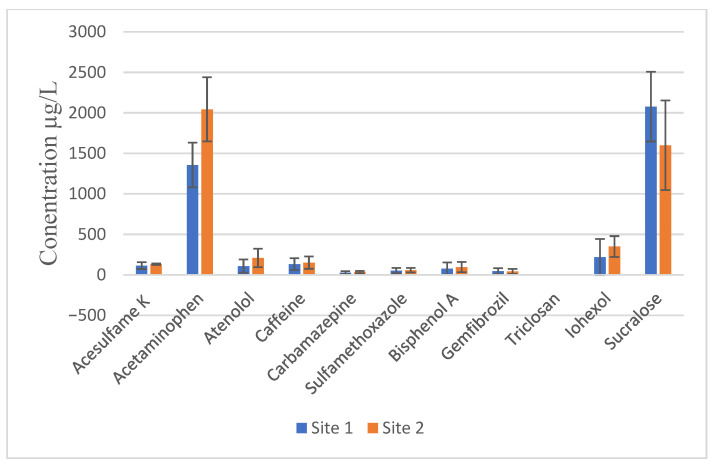
Summary of mean concentration levels of emerging contaminants from upstream sites (1 and 2). The error bars represent the standard deviation of replicate water samples collected at each site.

We conducted quantitative analysis using SPSS Version 27, performing descriptive analyses. Among the 16 emerging contaminants analyzed (Table 4), acetaminophen and sucralose were the most abundant, particularly at the upstream sites (Sites 1 and 2). Mean acetaminophen concentrations ranged from 1357 to 2044 µg/L, while sucralose ranged from 1600 to 2078 µg/L (Figure 1). Other emerging contaminants detected at the upstream sites included ioxehol (219–350 µg/L), caffeine (133–150 µg/L), acesulfame K (114–131 µg/L), and atenolol (108–209 µg/L). Bisphenol A was detected at 77–95 µg/L, sulfamethoxazole at 52–57 µg/L, and gemfibrozil at 42–45 µg/L. Several compounds including triclosan, 2,4-D (2-(2,4-dichlorophenoxy) acetic acid), atrazine, and its metabolites (desethyl atrazine, deisopropyl atrazine, and desethyl deisopropyl atrazine)—were not detected in any samples (Figure 2).

**Figure 3:**
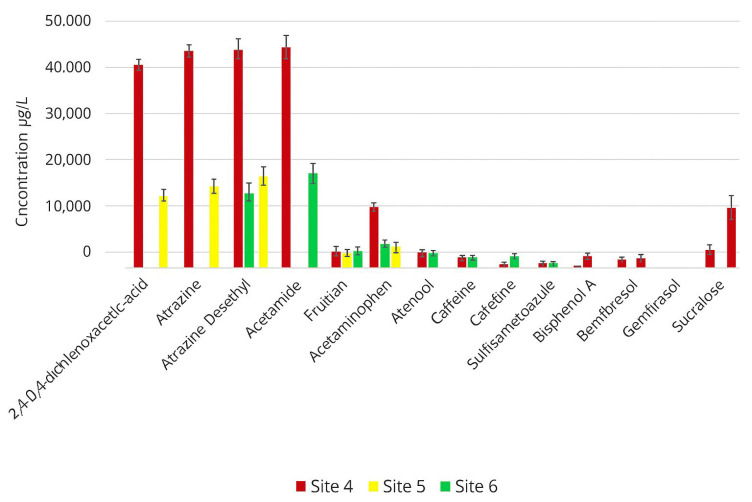
Summary of mean concentration levels of emerging contaminants from downstream sites (3, 4, and 5). The error bars represent the standard deviation of replicate water samples collected at each site.

At the downstream sites (Sites 3, 4, and 5), the mean concentrations of all detected emerging contaminants were higher than those observed at the upstream locations. The concentration ranges for the contaminants detected were as follows: 2,4-D (2-(2,4-dichlorophenoxy)acetic acid), 8715–34,800 µg/L; atrazine, 3838–9725 µg/L; atrazine desethyl, 1120–4300 µg/L; atrazine deisopropyl, 1100–4100 µg/L; and atrazine desethyl deisopropyl (DEDI), 1150–4310 µg/L. Additional contaminants included acesulfame K (134–525 µg/L), acetaminophen (2395–8177 µg/L), atenolol (305–835 µg/L), caffeine (88–140 µg/L), sulfamethoxazole (79–230 µg/L), bisphenol A (125–381 µg/L), gemfibrozil (50–170 µg/L), iohexol (112–146 µg/L), and sucralose (720–6400 µg/L). Triclosan was not detected at any downstream site. Among all downstream locations, Site 3 exhibited the highest mean concentrations for the detected contaminants (Figure 3).

## 4. Discussion

### 4.1. Spatial Distribution of Emerging Contaminants and Land-Use Influences

A clear spatial pattern was observed in the distribution of emerging contaminants upstream and downstream of the Detroit wastewater treatment plant (DWWTP). Of the sixteen contaminants analyzed, ten were consistently detected across all five sampling sites, reflecting their persistence and widespread presence throughout the Detroit River watershed. Higher concentrations downstream are likely due to increased urban anthropogenic sources, like agricultural runoff, contaminated overland flow, landfill leachate, and effluent from combined sewer overflows (CSOs). These findings indicate that urban and industrial development has led to significant anthropogenic impacts in the Great Lakes Basin watershed. Additionally, the amount and different varieties of emerging contaminants can be enhanced by pharmaceutical usage and variations in wastewater treatment efficiency [10,29,30].

The highest concentrations of emerging contaminants were observed at downstream Site 3, located near the Rouge River, a major tributary of the Detroit River. Both the Detroit and Rouge Rivers have historically been exposed to a large amount of industrial pollution and were reported as Areas of Concern by the International Joint Commission in 1985 [31,32]. The river corridors are heavily industrialized, with surrounding steel mills, petroleum refineries, power plants, and chemical manufacturing facilities. It has been reported that there are numerous effects on aquatic organisms and a lot of anthropogenic effects in the water due to urban runoff and industrial discharges in these rivers have contributed to elevated levels of emerging contaminants, leading to degraded water quality and posing risks to aquatic organisms [31,32].

During the 1960s the Detroit and Rouge Rivers were among the most polluted aquatic ecosystems ([33]) due to extensive anthropogenic pollution from unregulated oil spills, industrial and municipal discharges, inefficient wastewater treatment processes, and untreated effluents from combined sewer overflows (CSOs) [34,35]. Much of the Detroit and Rouge River watershed is occupied by residential, commercial, industrial, and agricultural land uses, all of which contribute to pollutant loading. In addition, the growing urban population within the Detroit Basin watershed has intensified anthropogenic pressures, further exacerbating water quality degradation.

The Detroit River watershed has experienced increased urbanization and industrial activity; consequently, the downstream sites exhibited frequent detection of numerous emerging contaminants, including 2,4-D (2-(2,4-dichlorophenoxy)acetic acid), atrazine and its transformation products (atrazine desethyl, atrazine deisopropyl, and atrazine desethyl deisopropyl [DEDI]), as well as acesulfame K, acetaminophen, atenolol, caffeine, sulfamethoxazole, bisphenol A, gemfibrozil, iohexol, and sucralose. Previous studies have demonstrated that many of these compounds persist in aquatic environments due to their high-water solubility and resistance to degradation, allowing them to remain in the water column and be transported throughout the watershed [36,37].

### 4.2. Pharmaceuticals

Increased concentrations of sulfamethazine and iohexol have been reported at downstream sites due to incomplete degradation [38]. This may lead to bioaccumulation and prolonged exposure to aquatic organisms. In our study, acetaminophen concentrations were unexpectedly higher at the upstream site compared to downstream locations, suggesting the influence of non-point sources such as domestic wastewater inputs or leakage from septic systems. Previous studies have reported elevated concentrations of acetaminophen, commonly used as an analgesic and antipyretic in rivers and streams across the United States. In addition, cardiovascular pharmaceuticals such as atenolol (a beta-blocker) and gemfibrozil (a lipid regulator) were consistently detected at higher concentrations downstream, indicating the influence of urban wastewater discharges due to contaminant loading. Although the observed concentrations of atenolol and gemfibrozil were lower than those reported in previous studies, their persistence and bioactivity have been reported to affect aquatic ecosystems. Iohexol is a non-ionic, water-soluble, iodine-based contrast agent widely used in medical imaging procedures, particularly in computed tomography (CT) and angiography. The detection of iohexol at both upstream and downstream sampling sites suggests active and ongoing inputs from medical waste discharges, highlighting the influence of urban healthcare activities on surrounding aquatic systems. In our study, iohexol was detected at both upstream and downstream sampling locations, consistent with previous reports documenting elevated concentrations downstream of wastewater treatment plants.

### 4.3. Industrial and Consumer Chemicals

Bisphenol A (BPA), an emerging contaminant classified as an alkylphenolic compound, was detected at both upstream and downstream sampling sites, with higher concentrations observed downstream. BPA is widely used in the manufacture of consumer and industrial products, including baby bottles, dental materials, food and beverage containers, and building materials [39].

Similarly, caffeine, a widely used stimulant and anthropogenic marker of domestic wastewater, was detected at elevated concentrations downstream. These levels are consistent with previous findings from the Canadian side of the Detroit River and other urban watersheds [2,4,5,40,41,42,43].

### 4.4. Agricultural Chemical

Atrazine, a widely used herbicide, remains one of the most frequently detected pesticides in aquatic environments, particularly in regions with intensive agricultural activity [2,4,6,37,44,45]. Its high detection frequency is often attributed to runoff from farmlands, which serves as a major source of pesticide loading into adjacent waterways. Studies from agricultural sites in Chatham and London, Ontario, have demonstrated that watershed runoff significantly influences atrazine concentrations in surface waters, highlighting the hydrological connectivity between agricultural practices and downstream water quality [37]. In the Detroit River watershed, the detection of atrazine at both upstream and downstream sites indicates widespread agricultural inputs contributing to the cumulative contaminant burden in the river system.

### 4.5. Antibiotics

Similarly, sulfamethoxazole, an antibiotic detected at both upstream and downstream sites, represents a growing environmental challenge. Antibiotics are particularly concerning due to their persistence, incomplete metabolism, and ease of dispersion in aquatic systems [46].

### 4.6. Antimicrobial Compound

In our study, triclosan was not detected at either the upstream or downstream sites near the wastewater treatment plant. This absence likely reflects regulatory actions, as triclosan has been restricted in personal care products since 2016 due to concerns over human health and ecotoxicological effects, including endocrine disruption and potential carcinogenicity [47]. The lack of detection contrasts with earlier studies that reported triclosan in surface waters [42], highlighting the positive impact of regulatory measures on reducing environmental contamination.

### 4.7. Wastewater Treatment Plant (WWTP) Removal Efficiency

The findings for sulfamethazine and iohexol underscore the urgent need to enhance wastewater treatment technologies and implement integrated watershed management strategies to reduce contaminant inputs and protect both ecological integrity and human health in the Detroit River system. Iohexol, a radiocontrast agent, is metabolically stable and largely excreted unchanged following medical use, allowing it to readily enter wastewater streams and persist into receiving surface waters [4,48]. Its consistent detection reflects both its widespread usage and limited removal during conventional treatment processes.

Similarly, atenolol, a beta-blocker, is primarily excreted in its parent form and exhibits limited biodegradation, contributing to its persistence in aquatic environments [4,42]. Although measured concentrations provide valuable insight, they may underestimate the ecological risk posed by compounds such as gemfibrozil, which can exist as bioactive metabolites with similarly low removal efficiencies. The frequent occurrence of atenolol and gemfibrozil in urban watersheds is largely driven by high pharmaceutical consumption and the proximity of wastewater discharge points [4,40,42]. These observations highlight the importance of incorporating transformation products and metabolites into environmental monitoring frameworks and ecological risk assessments ([4,49] Carbamazepine, a widely used anticonvulsant, was detected at both upstream and downstream sites, further demonstrating its persistence and resistance to conventional wastewater treatment. Its chemical stability and high water solubility limit its removal during treatment processes, allowing it to persist in effluents and surface waters [28,42,44,50]. The continued presence of such compounds highlights the vulnerability of urban river systems, where treated and untreated wastewater inputs contribute to chronic exposure of aquatic organisms to bioactive contaminants.

The co-occurrence of compounds such as iohexol and carbamazepine exemplifies the broader challenge of pharmaceutical persistence in urban watersheds and reinforces the need for improved treatment technologies, comprehensive monitoring of transformation products, and evaluation of cumulative ecological risks. In addition, although caffeine is not highly persistent, its repeated detection indicates continuous inputs from untreated or partially treated wastewater sources, including sewage leaks, combined sewer overflows, and stormwater runoff. Alongside bisphenol A (BPA), caffeine serves as an indicator of ongoing anthropogenic contamination, highlighting the complex mixtures of bioactive pollutants entering aquatic systems and the need for improved wastewater management practices.

Despite the persistence of many contaminants, wastewater treatment effluents may exhibit lower concentrations due to dilution, increased distance from discharge points, and partial removal during treatment [51]. The absence of triclosan in the sampled sites suggests that regulatory actions and policy interventions can effectively reduce environmental concentrations of certain contaminants, thereby supporting ecosystem health.

Previous studies consistently report incomplete removal of emerging contaminants in WWTPs. Reference [52] detected atenolol in all effluent samples analyzed, with removal primarily attributed to microbial biodegradation rather than sorption, due to its low partition coefficient (Kd < 500 L/kg). Similarly, Reference [53] observed atenolol in both primary and final effluents, confirming its persistence throughout treatment stages. Other pharmaceuticals, including gemfibrozil, bezafibrate, clofibric acid, antibiotics, and carbamazepine, have also been detected in influents and effluents of treatment plants in the Great Lakes region, indicating incomplete removal during standard processes [21].

The ecological implications of this persistence are significant. Continuous discharge of these compounds into aquatic environments can disrupt physiological processes in aquatic organisms, impair reproduction, alter microbial community composition, and contribute to long-term contaminant accumulation in freshwater systems.

In contrast, some compounds, such as caffeine and cotinine, are relatively well removed during wastewater treatment despite high influent concentrations, resulting in low levels in effluents [21]. However, other pharmaceuticals including carbamazepine, fluoxetine, ibuprofen, and atorvastatin have been detected in surface waters and even drinking water sources such as Lake Huron, reflecting their resistance to conventional treatment [17].

Biological treatment processes in WWTPs remove contaminants primarily through biodegradation and sorption [22]. Biodegradation may occur via direct microbial metabolism or co-metabolism, where other compounds serve as energy sources. Sorption, on the other hand, depends on physicochemical properties such as hydrophobicity and charge interactions, with more hydrophobic compounds generally exhibiting greater removal efficiency [22,54]. Consequently, highly water-soluble compounds with low hydrophobicity, such as sulfamethoxazole, tend to exhibit poor removal and persist in effluents.

Removal efficiencies vary widely among WWTPs, typically ranging from 20% to 99%, depending on treatment type, operational conditions (e.g., sludge retention time, hydraulic retention time, pH, temperature), and contaminant properties [4,54,55,56]. Among these factors, treatment configuration and compound characteristics are the most influential. Notably, highly persistent compounds such as carbamazepine may exhibit removal efficiencies below 20%, allowing them to persist in treated effluents and enter receiving waters.

Overall, the persistence of emerging contaminants in wastewater effluents presents a significant environmental challenge. Their continued release into aquatic systems raises concerns about bioaccumulation, disruption of ecosystem processes, and chronic exposure risks to aquatic organisms. These findings emphasize the critical need for advanced treatment technologies, improved monitoring strategies, and integrated watershed management approaches to safeguard freshwater resources.

Collectively, these findings highlight the urgent need to enhance wastewater treatment technologies and develop comprehensive monitoring frameworks to mitigate pharmaceutical pollution and its cascading impacts on aquatic ecosystems and overall water quality.

### 4.8. Ecological and Human Health Implications

Exposure to pharmaceuticals such as acetaminophen has been associated with a range of adverse biological effects in aquatic organisms, including transgenerational impacts, neurobehavioral toxicity, reproductive impairment, and oxidative stress [4,57,58,59]. These effects raise significant ecological concerns, as they have the potential to alter population dynamics, disrupt trophic interactions, and ultimately affect overall ecosystem functioning.

In contrast, despite the widespread detection of iohexol in aquatic environments, limited information is available regarding its ecotoxicological effects and environmental transformation pathways [4,48]. This lack of data represents a critical knowledge gap, making it difficult to fully assess its long-term persistence and ecological risks. Consequently, targeted monitoring and toxicity studies are needed to better understand the environmental behavior and biological impacts of iohexol.

Carbamazepine has been widely documented to disrupt endocrine function and impair reproduction in fish, posing risks to aquatic population health and food web stability [10,59]. Its frequent detection in surface waters reflects both its extensive usage and resistance to degradation during wastewater treatment processes. Similarly, bisphenol A (BPA) is of particular concern due to its well-established estrogenic activity. Studies have shown that even at concentrations below 1 µg/m^3^, BPA can interfere with endocrine signaling in aquatic organisms [60]. At environmentally relevant concentrations, BPA has been linked to oxidative stress, neurotoxicity, and reproductive impairments in both fish and invertebrates [61]. The elevated downstream concentrations observed in this study likely reflect inputs from wastewater effluents and urban runoff, underscoring its bioactive nature and ecological significance.

Caffeine, although often considered relatively benign, can also exert measurable biological effects. Experimental studies have demonstrated that caffeine exposure may induce developmental toxicity, behavioral alterations, and anxiety-like responses in aquatic organisms, particularly when present in mixtures with other contaminants such as pharmaceuticals or artificial sweeteners [4,41,62]. These findings highlight the importance of considering mixture toxicity when evaluating ecological risks.

Atrazine, a widely used herbicide, represents a persistent and mobile contaminant in aquatic systems. Its presence has been associated with endocrine disruption, reproductive abnormalities, and declines in biodiversity, raising concerns about long-term ecosystem stability. Due to its agricultural origins, atrazine also reflects the influence of non-point source pollution on watershed health.

Sulfamethoxazole and other antibiotics present an additional layer of ecological and public health concern. Once released into aquatic environments, these compounds can promote the selection and proliferation of antibiotic-resistant bacteria, facilitating the spread of antibiotic resistance genes (ARGs) [63]. This process has far-reaching implications, as it contributes to the global challenge of antimicrobial resistance. Furthermore, antibiotics may disrupt wastewater treatment processes by inhibiting microbial communities responsible for organic matter degradation and nutrient cycling. Ecologically, such disruptions can alter microbial food webs, impair nutrient dynamics, and reduce ecosystem resilience.

From a human health perspective, exposure to antibiotic-contaminated water through recreational activities or drinking water sources may result in adverse outcomes, including cardiac arrhythmias, immune dysfunction, liver toxicity, and bone marrow suppression [64].

Collectively, the occurrence of contaminants such as atrazine and sulfamethoxazole highlights the combined pressures of agricultural and urban inputs on the Detroit River watershed. These findings emphasize the urgent need for integrated watershed management strategies that reduce agrochemical runoff, improve wastewater treatment efficiency, and address the cumulative impacts of chemical mixtures to protect both ecosystem integrity and human health.

### 4.9. Implications for Monitoring and Management

These findings indicate that water quality in the Detroit River is strongly influenced by the presence and distribution of emerging contaminants, particularly in relation to wastewater treatment plant (DWWTP) inputs. The upstream site consistently exhibited lower mean concentrations of all detected contaminants compared to downstream locations, highlighting the influence of treated effluent and other anthropogenic sources on contaminant loading. Elevated downstream concentrations likely reflect cumulative inputs from urban and industrial discharges, as well as incomplete removal during wastewater treatment.

Removal efficiencies for emerging contaminants in wastewater treatment plants vary widely, typically ranging from 20% to 99%, depending on compound-specific physicochemical properties and treatment technologies [4,27]. Compounds such as caffeine and atenolol often demonstrate relatively high removal efficiencies (>90%), whereas more persistent and hydrophilic substances, including carbamazepine and sucralose, frequently exhibit removal rates below 30%. As a result, even with dilution effects downstream, the persistence of these recalcitrant compounds contributes to their continued detection at elevated concentrations. Conversely, the absence of certain contaminants at upstream sites may reflect both lower anthropogenic inputs and reduced environmental persistence.

Characterizing the occurrence and concentration gradients of emerging contaminants provides critical insight into both treatment performance and environmental transport pathways. These data are particularly valuable when evaluated against established environmental and regulatory benchmarks, including predicted no-effect concentrations (PNECs), World Health Organization (WHO) guidelines, and U.S. Environmental Protection Agency (EPA) drinking water standards. In this study, concentrations of several contaminants—including acetaminophen (1357–8177 µg/L), bisphenol A (77–381 µg/L), and sucralose (1600–6400 µg/L)—exceeded ecological and human health thresholds by one to two orders of magnitude. Such exceedances underscore the potential risks to aquatic organisms and raise concerns regarding the safety and sustainability of drinking water resources.

These findings have important implications for both monitoring and management strategies. First, they highlight the need for expanded monitoring programs that include not only parent compounds but also transformation products and contaminant mixtures, which may exhibit additive or synergistic effects. Second, they emphasize the importance of upgrading wastewater treatment infrastructure to incorporate advanced treatment technologies—such as ozonation, activated carbon adsorption, and membrane filtration—that are more effective at removing persistent contaminants. Finally, the results support the adoption of integrated watershed management approaches that address both point and non-point pollution sources, including urban runoff, agricultural inputs, and combined sewer overflows.

## 5. Conclusions

This study demonstrates the widespread occurrence and spatial variability of multiple classes of emerging contaminants in the Detroit River, particularly in relation to the Detroit wastewater treatment plant (DWWTP). Of the sixteen compounds analyzed, several pharmaceuticals (e.g., acetaminophen, atenolol, sulfamethoxazole, and carbamazepine), artificial sweeteners (sucralose, acesulfame K), industrial and consumer chemicals (bisphenol A), stimulants (caffeine), pesticides (atrazine, 2,4-D, and atrazine transformation products), and contrast agents (iohexol) were detected at both upstream and downstream sites, with consistently higher concentrations observed downstream.

Notably, compounds such as acetaminophen and sucralose were among the most abundant across sites, while atrazine and its metabolites (desethyl atrazine, deisopropyl atrazine, and DEDI atrazine), as well as 2,4-D, were detected at substantially elevated concentrations downstream, highlighting the combined influence of agricultural runoff and wastewater discharge. The frequent detection of atenolol, gemfibrozil, sulfamethoxazole, and carbamazepine further underscores the persistence of pharmaceuticals that are incompletely removed during conventional wastewater treatment processes.

The presence of bisphenol A and caffeine at elevated downstream concentrations indicates ongoing inputs from urban and industrial sources, while the detection of iohexol reflects contributions from medical and healthcare activities. In contrast, the absence of triclosan suggests that regulatory actions can effectively reduce environmental concentrations of certain contaminants.

Although some measured concentrations fall below current regulatory thresholds, the consistent detection of bioactive compounds such as sulfamethoxazole, bisphenol A, carbamazepine, and atrazine raises concerns regarding chronic exposure, mixture effects, endocrine disruption, and the potential development of antibiotic resistance in aquatic environments. These risks are particularly important given the persistence, bioactivity, and continuous input of these contaminants into surface waters.

Overall, this study highlights the need for targeted monitoring frameworks that prioritize specific high-risk compounds and contaminant classes identified in this work, rather than relying on generalized assessments of “emerging contaminants.” Furthermore, the findings emphasize the importance of advancing wastewater treatment technologies to improve the removal of persistent and hydrophilic compounds such as carbamazepine, sucralose, and sulfamethoxazole, as well as incorporating transformation products and metabolites into routine monitoring.

Future research should focus on quantifying the ecological and human health risks associated with long-term, low-level exposure to mixtures of contaminants, particularly those identified in this study, and on developing integrated watershed management strategies to reduce inputs from urban, agricultural, and healthcare sources.

## Figures and Tables

**Table 1:** Description of sampling locations.

Site Code	Location Name	Latitude (N)	Longitude (W)
1	Clinton River Mouth	42°35′40.7″	82°46′01.6″
2	Northeast Belle Isle	42°35′17.1″	82°95′94.7″
3	Southwest Belle Isle	42°33′35.0″	82°98′64.2″
4	Rouge River Mouth	42°27′52.1″	83°11′07.6″
5	Detroit River International Wildlife Refuge/Trenton Channel	42°09′97.8″	83°18′26.0″
★	Detroit Wastewater Treatment Plant (DWWTP)	—	—

**Table 2:** Physicochemical parameters measured at each sampling site and corresponding measurement techniques.

Parameter	Unit	Measurement Technique/Instrument
Water temperature	°C	Digital water quality flow meter; GPS-integrated sensor (Garmin)
Total dissolved solids (TDS)	ppm	Digital water quality flow meter; GPS-integrated sensor (Garmin)
pH	—	Digital water quality flow meter; GPS-integrated sensor (Garmin)
Turbidity	NTU	Digital water quality flow meter; GPS-integrated sensor (Garmin)
Electrical conductivity (EC)	µS/cm	Digital water quality flow meter; GPS-integrated sensor (Garmin)
Water depth	m	GPS-integrated depth sensor (Garmin)
Precipitation (30-day total)	mm	NOAA weather station records

**Table 3:** Physicochemical parameters (pH, temperature, turbidity, and conductivity) of surface water collected from sampling Sites 1–5 near the Detroit wastewater treatment plant. Data adapted from [29].

Site	pH	Temp (°C)	Turbidity (ppm)	Conductivity (μS/cm)
Site 1 (upstream)	7.80	29.00	208.00	435.00
Site 2 (upstream)	8.00	25.90	676.00	929.00
Site 3 (downstream)	7.90	22.80	378.00	526.00
Site 4 (downstream)	9.00	19.00	198.00	288.00
Site 5 (downstream)	8.39	17.90	170.00	236.00
Range	7.80–9.00	17.90–29.00	170.00–676.00	236.00–929.00

**Table 4:** Emerging contaminants that were analyzed from Sites 1–5.

Emerging Contaminants	Chemical Structure	Molecular Formula	Molecular Weight g/mol	LogKow	pKa
2,4-D 2-(2,4-dichlorophenoxy)acetic acid	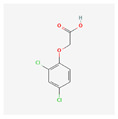	C_8_H_6_Cl_2_O_3_	221.03	2.81	2.64
Atrazine	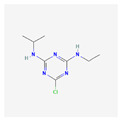	C_8_H_14_ClN_5_	215.68	2.61	1.60
Atrazine Desethyl	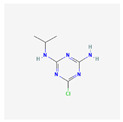	C_6_H_10_ClN_5_	187.63	1.5	3.79
Atrazine Deisopropyl	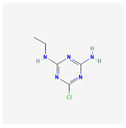	C_5_H_8_ClN_5_	173.60	1.1	3.85
Atrazine Desethyl Deisopropyl (DEDI Atrazine)	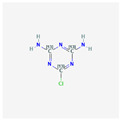	C_3_H_4_ClN_5_	148.53		
Acesulfame K	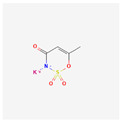	C_4_H_4_KNO_4_S	201.24	1.41	2.0
Acetaminophen	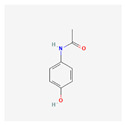	C_8_H_9_NO_2_	151.16	0.5	9.38
Atenolol	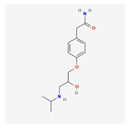	C_14_H_22_N_2_O_3_	266.34	0.2	9.6
Caffeine	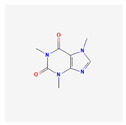	C_8_H_10_N_4_O_2_	194.19	−0.1	14
Carbamazepine	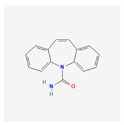	C_15_H_12_N_2_O	236.27	2.5	13.9
Sulfamethoxazole	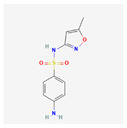	C_10_H_11_N_3_O_3_S	253.28	0.9	0.89
Bisphenol A	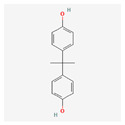	C_15_H_16_O_2_	228.29	3.3	9.6
Gemfibrozil	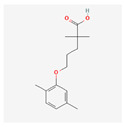	C_15_H_22_O_3_	250.33	3.8	4.5
Triclosan	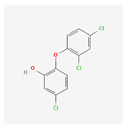	C_12_H_7_Cl_3_O_2_	289.5	5	7.9
Iohexol	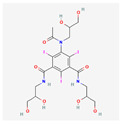	C_19_H_26_I_3_N_3_O_9_	821.1	−3	11.73
Sucralose	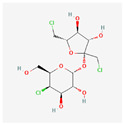	C_12_H_19_Cl_3_O_8_	397.6	−1.5	11.91

Chemical structure, molecular formula, molecular weight, LogKow and pKa based on https://pubchem.ncbi.nlm.nih.gov/ (accessed on 15 April 2026).

## Data Availability

Data cannot be made publicly available; readers should contact the corresponding author for details.
